# Pyogenic liver abscess as a late complication after embolization of a hepatic adenoma

**DOI:** 10.1590/0100-3984.2017.0143

**Published:** 2019

**Authors:** Thiago Franchi Nunes, Fabio Colagrossi Paes Barbosa, Tiago Kojun Tibana, Edson Marchiori

**Affiliations:** 1 Universidade Federal de Mato Grosso do Sul (UFMS), Campo Grande, MS, Brazil.; 2 Universidade Federal do Rio de Janeiro (UFRJ), Rio de Janeiro, RJ, Brazil.

*Dear Editor,*

A 28-year-old woman who had been taking an oral contraceptive (OC) for 10 years underwent magnetic resonance imaging (MRI), which showed multiple tumors. The tumors were isoattenuating in the pre-contrast phase, showed homogeneous enhancement in the arterial phase, and were isoattenuating again (with enhancement comparable to that of the liver parenchyma) in the subsequent phases (Figure 1A). The pathology study confirmed the diagnosis of adenoma. The largest tumor, measuring approximately 10 cm, was compressing the inferior vena cava, making the surgical approach difficult, and presented a high risk of intraoperative complications. We opted for arterial embolization, which was performed successfully, and no vascularization was observed on a follow-up computed tomography (CT) scan. Seven months later, the patient returned with complaints of abdominal pain, daily fever, and weight loss. At that time, an MRI scan showed a collection, consistent with abscess, near the site of the adenoma (Figure 1B). Ultrasound-guided percutaneous drainage was performed, and 800 mL of purulent secretion were drained (Figure 1C). After a seven-day course of antibiotic therapy with saline lavage of the abscess, the patient progressed to complete resolution of the condition. A follow-up MRI scan, acquired six months after the percutaneous drainage, confirmed that the treatment had been successful (Figure 1D).

**Figure 1:**
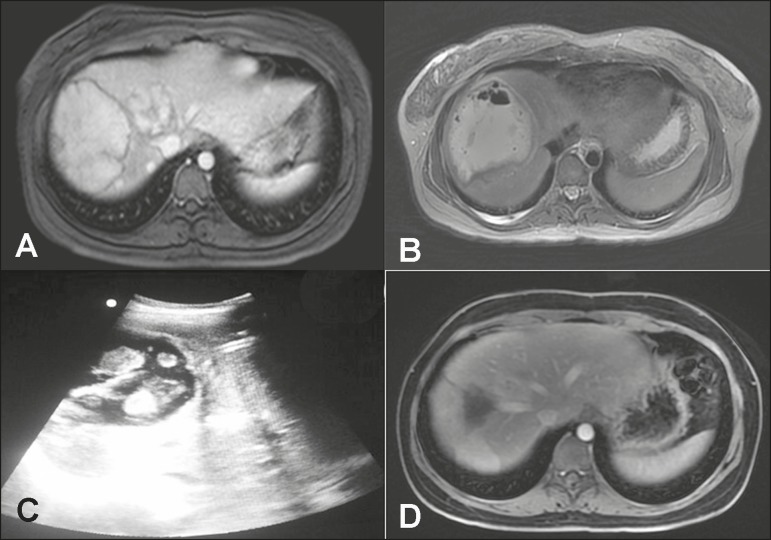
**A:** Abdominal MRI performed prior to embolization, showing a hypervascular lesion measuring approximately 10 cm, the histological analysis of which showed hepatic adenoma that tested positive for the tumor marker beta-catenin. **B:** Abdominal MRI scan, acquired at six months after embolization, revealing gaseous contents within the treated lesion. **C:** Ultrasound-guided percutaneous drainage of the hepatic collection/abscess. **D:** Follow-up abdominal MRI scan, acquired at six months after the percutaneous treatment of the hepatic abscess.

Hepatocellular adenoma (HCA) is a rare benign tumor of the liver that is commonly seen in women of reproductive age and is associated with the use of OCs^(^^1^^)^. The annual incidence of HCA is 3-4 cases/100,000 women who have used OCs for an extended period of time. Approximately 25% of patients with HCA experience bleeding, the risk of which increases in parallel with an increase in tumor diameter. Malignant transformation occurs in up to 4% of all cases of HCA^(^^2^^,^^3^^)^. The risk of malignant transformation also increases as tumor diameter increases, and excision is generally recommended for tumors that are still larger than 5 cm in diameter after OC discontinuation^(^^4^^)^.

Transarterial embolization (TAE) is widely used for the treatment of bleeding adenomas and can be performed before elective surgery to reduce intraoperative blood loss. In HCA patients, TAE can reduce the size of large adenomas, multiple adenomas, or adenomas that are in a surgically inaccessible location, in order to reduce symptoms and the risk of bleeding^(^^5^^-^^7^^)^. Given that the risk of malignant transformation is directly proportional to the size of the adenoma^(^^7^^)^, TAE can reduce this risk. However, the role of TAE as an elective therapy in HCA is unclear, because it is not known whether it reduces the risk of hemorrhage or malignant transformation of residual HCA, despite reports of a reduction in tumor size^(^^8^^)^.

In patients with HCA, the most common complication of TAE is post-embolization syndrome, followed by transient renal insufficiency and cyst formation^(^^8^^)^. In the case presented here, the patient evolved to late liver abscess after embolization of the adenoma. To our knowledge, there have been no previous reports of this complication. The treatment of pyogenic liver abscess includes intravenous antibiotic therapy and percutaneous drainage guided by ultrasound or CT.

Acute or elective TAE seems to be a safe procedure for the management of HCA. Because of its minimally invasive and parenchyma-preserving properties, together with its ability to reduce the size of tumors located at anatomical sites that make surgery difficult, elective TAE offers a reasonable alternative to surgery.

## References

[1] Edmondson HA, Henderson B, Benton B (1976). Liver-cell adenomas associated with use of oral
contraceptives. N Engl J Med.

[2] Søe KL, Søe M, Gluud C (1992). Liver pathology associated with the use of anabolic-androgenic
steroids. Liver.

[3] Ault GT, Wren SM, Ralls PW (1996). Selective management of hepatic adenomas. Am Surg.

[4] Rooks JB, Ory HW, Ishak KG (1979). Epidemiology of hepatocellular adenoma. The role of oral
contraceptive use. JAMA.

[5] Agrawal S, Agarwal S, Arnason T (2015). Management of hepatocellular adenoma: recent
advances. Clin Gastroenterol Hepatol.

[6] Nasser F, Affonso BB, Galastri FL (2013). Minimally invasive treatment of hepatic adenoma in special
cases. Einstein (Sao Paulo).

[7] Erdogan D, Busch OR, van Delden OM (2006). Management of spontaneous haemorrhage and rupture of
hepatocellular adenomas. A single centre experience. Liver Int.

[8] van Rosmalen BV, Coelen RJS, Bieze M (2017). Systematic review of transarterial embolization for
hepatocellular adenomas. Br J Surg.

